# Prospective study on the association between serum amino acid profiles and depressive symptoms among the Japanese working population

**DOI:** 10.1371/journal.pone.0256337

**Published:** 2021-08-17

**Authors:** Takako Miki, Masafumi Eguchi, Takeshi Kochi, Ami Fukunaga, Sanmei Chen, Akiko Nanri, Isamu Kabe, Tetsuya Mizoue

**Affiliations:** 1 Department of Epidemiology and Prevention, Center for Clinical Sciences, National Center for Global Health and Medicine, Tokyo, Japan; 2 Department of Health Administration, Furukawa Electric Corporation, Tokyo, Japan; 3 Department of Global Health Nursing, Graduate School of Biomedical and Nursing Sciences, Hiroshima University, Hiroshima, Japan; 4 Department of Food and Health Sciences, International College of Arts and Sciences, Fukuoka Women’s University, Fukuoka, Japan; 5 Kubota Corporation, Ibaraki, Japan; National Institute on Drug Abuse, UNITED STATES

## Abstract

**Objective:**

Accumulating evidence suggests that amino acids, particularly tryptophan and glutamate, play an important role in the pathology of depression, but prospective epidemiologic data on this issue is scarce. We examined the association between circulating amino acids and the risk of depressive symptoms in a Japanese working population.

**Methods:**

Participants were 841 workers who were free from depressive symptoms and provided blood at baseline and completed 3-yr follow-up survey. 30 varieties of amino acid concentrations in serum were measured using liquid chromatography/mass spectrometry. Depressive symptoms were defined using the Center for Epidemiologic Studies Depression Scale. Logistic regression was used to calculate the odds ratios of depressive symptoms according to serum amino acids with adjustment for lifestyle factors.

**Results:**

A total of 151 (18.0%) workers were newly identified as having depressive symptoms at the follow-up. Baseline tryptophan and glutamate concentrations in serum were not appreciably associated with the risk of depressive symptoms. Risk of depressive symptoms tended to increase with increasing arginine concentrations; the multivariable-adjusted odds ratio for the highest versus lowest tertile of serum arginine was 1.65 (95% confidence interval: 0.96–2.83; P for trend = 0.07). No clear association was found for other amino acids.

**Conclusions:**

Results of the present study do not support a significant role of circulating amino acids in the development of depressive symptoms among Japanese.

## Introduction

Depression is a common mental health issue, which reduces productivity at work, lowers the quality of life, as well as increases mortality [1]. Accumulating evidence suggests that dietary factors may be important for brain function and mental health [2]. For instance, changes in the serotonergic [3, 4], dopaminergic [5], noradrenergic [3, 4], and glutamatergic [6, 7] systems are suggested to be involved in depression. Amino acids can act as neurotransmitters in these systems or as precursors of neurotransmitters [8]; serotonin neurotransmitter is derived from the amino acid tryptophan [8]. In addition, catecholamine neurotransmitters, such as dopamine and noradrenaline, are synthesized from tyrosine or its precursor phenylalanine [8]. A shortage or impaired processing of these amino acids may result in decreased levels of mood-regulating neurotransmitters, which are thought to be involved in depression [8]. In contrast, when present in excess, excitatory amino acids such as of glutamate are known to act as potent neuronal excitotoxins, triggering neurotoxicity in pathology of depression [6], and increased concentrations of excitatory amino acids may occur in the brain as a result of peripheral alteration of the availability or metabolism of these amino acids [9].

Consistent with these pathophysiologic concepts, epidemiological evidence points to a link between circulating amino acids and depression. Meta-analyses showed lower peripheral blood concentrations of tryptophan [10] and higher glutamate concentrations [11] in patients with depressive disorder compared with controls. Regarding other amino acids, evidence is limited and inconsistent. For instance, one study found that plasma tyrosine and phenylalanine concentrations were significantly decreased in depressive patients compared with healthy controls [12], whereas other studies found no significant differences [13, 14] or even conversely a significant increase in patients with depression compared with controls [15, 16]. Likewise, excitatory amino acids, such as aspartate (aspartic acid) [13] and arginine [17], were reported to be significantly higher in depressive individuals than in controls, although conflicting data exist concerning these amino acids [18].

Several issues remain unsettled. First, all previous studies on this issue [10–18] were cross-sectional and thus subject to reverse causality. This is of particular concern for studies comparing amino acid concentrations between depressive patients on medication and healthy controls [10, 12, 18]; the observed changes in amino acid concentrations may be attributable to the effects of medication, such as antidepressants, on circulating amino acids [19, 20]. Second, from a prevention perspective, identifying the factors predictive of depressive symptoms in free-living people is a priority issue. However, the majority of previous studies have been carried out in clinical settings [10–16, 18], and only two small studies (n<120) were performed in the community-dwelling elderly [17, 21].

The aim of the present study was to examine prospective the association between serum amino acid profiles at baseline and the onset of depressive symptoms in a cohort of workers in Japan. The primary hypothesis was that higher concentrations of tryptophan are associated with a decreased risk of depressive symptoms, whereas higher concentrations of glutamate are associated with an increased depressive risk.

## Materials and methods

### Study procedure and participants

As part of the Japan Epidemiology Collaboration on Occupational Health Study, a nutritional epidemiological survey named the Furukawa Nutrition and Health Study was conducted during periodic health examinations of workers from two locations of a manufacturing company and its affiliates. Details of the study procedure were described elsewhere [22, 23]. The survey has been performed every three years since 2012 in A factory and 2013 in B factory. In the present study, we treated the 2015–16 survey, when serum amino acids were measured, as the baseline and the 2018–19 survey as the follow-up for the assessment of depressive symptoms.

Prior to the health checkup, we asked all workers to participate in the survey and fill out two questionnaires, one specifically designed for diet and the second for overall health-related lifestyle. We asked participants to donate 7-mL venous blood for study. We obtained health checkup data, including the results of anthropometric and biochemical measurements and disease history.

The protocol for the study was approved by the ethics committee of the National Center for Global Health and Medicine, Japan. Prior to each survey, written informed consent was obtained from all participants.

### Participants

**Fig 1:**
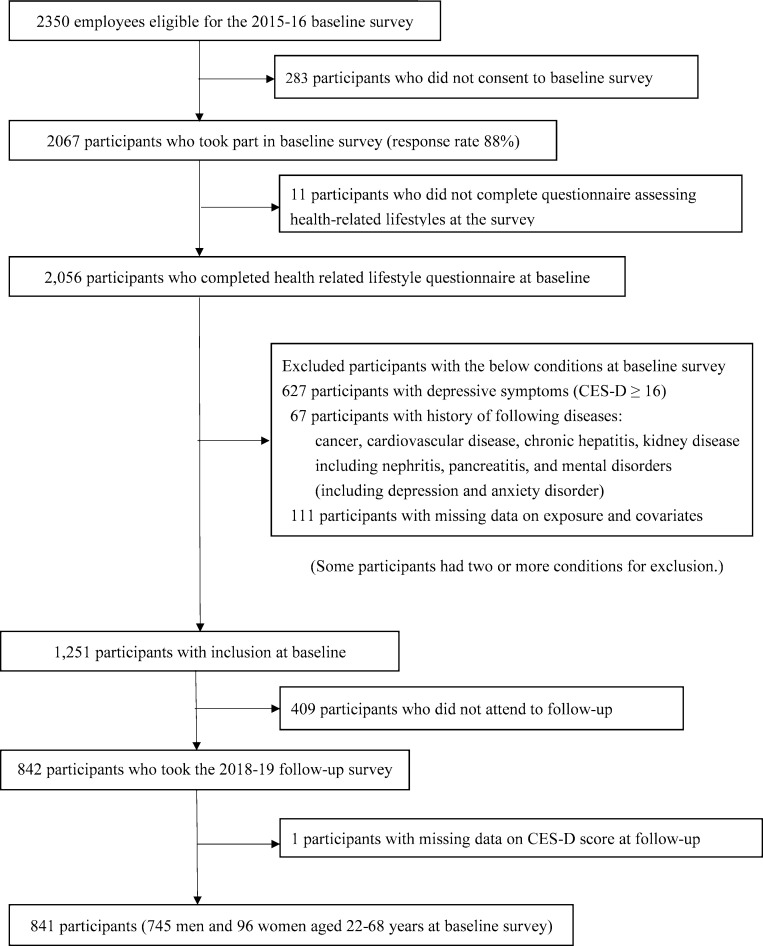
Flowchart of study protocol. Abbreviation: CES-D, Center for Epidemiologic Studies Depression Scale.

Of 2350 eligible participants at the baseline survey, 2067 employees participated (response rate: 88%). Of these, 2056 participants completed the questionnaire assessing their health-related lifestyle at the baseline survey. We initially excluded 627 participants with depressive symptoms (Center for Epidemiologic Studies Depression [CES-D] ≥ 16) and 67 participants with a history of the following diseases diagnosed by a physician: cancer (n = 23), cardiovascular diseases (n = 19), chronic hepatitis (n = 2), kidney diseases (e.g. nephritis) (n = 6), pancreatitis (n = 2), and mental disorders, including depression and neurotic disorder (n = 16). Some participants had two or more conditions meriting exclusion. We made these exclusions to eliminate potential reverse causality; dietary changes as a result of such diseases may have affected serum amino acid concentrations. We then excluded 111 with missing data on serum amino acids and covariates at the baseline survey. Of the remaining 1251 participants, 841 (745 men and 96 women aged 22–68 years) participated in the follow-up survey and filled out the study questionnaire (**Fig 1**).

### Assessment of depressive symptoms

Depressive symptoms were assessed using the Japanese version [24] of the CES-D scale [25], which was incorporated into the lifestyle questionnaire at both baseline and 3-yr surveys. This scale consists of 20 items covering the symptoms of depression, including depressed mood, guilt or worthlessness, helplessness or hopelessness, psychomotor retardation, loss of appetite, and sleep disturbance experienced during the preceding week. Each item is scored on a scale of 0–3 according to the frequency of the symptom and summed to obtain the total CES-D score, which ranges from 0 to 60. Participants with a CES-D score ≥16 were considered as having depressive symptoms. The criterion validity of the CES-D scale has been established in both Western [25] and Japanese [24] individuals. In a validation study for the Japanese version, a CES-D scale score cutoff of ≥16 presented a sensitivity of 88.2% and specificity of 84.8% [24].

### Amino acid measurement

Participants were instructed to receive the checkup after an overnight fast. Venous blood (7 ml) donated for the study was drawn into a vacuum tube and then transported in a cooler box to a laboratory. Blood was centrifuged for 15 min to separate serum, and the serum sample was stored at -80°C until the analyses were performed. Serum concentrations of amino acids were analyzed using liquid chromatography/mass spectrometry (LC/MS) at an external laboratory (SRL, Tokyo Japan). Serum samples deproteinized using acetonitrile were centrifuged twice at 12,000 rpm for 10 min, and then the supernatant was aliquoted. The aliquoted samples were then separated on an Inertsil ODS-3 column (2.1×100 mm; GL Science, Tokyo, Japan) during the analysis. Amino acid concentrations were determined via an LC-MS 2020 system (Shimadzu Corp., Kyoto, Japan). We initially targeted 39 amino acid metabolites. Of these, 30 were detectable and eligible for final analyses (i.e. 3 branched-chain amino acids [BCAAs] [isoleucine, leucine, and valine], 2 aromatic amino acids [phenylalanine, tyrosine], 17 gluconeogenic amino acids and other amino acids [aspartic acid, asparagine, alanine, β-alanine, arginine, glycine, glutamate, glutamine, histidine, methionine, proline, serine, tryptophan, threonine, citrulline, ornithine, lysine, and cystine], and 7 intermediary organic acids [α-aminobutyric acid, β-aminoisobutyric acid, 1-methylhistidine, 3-methylhistidine, monoethanolamine, hydroxyproline, and taurine]). Concentrations of these metabolites were expressed in micromoles (μmol) per liter (L).

### Other variables

The survey questionnaire enquired about marital status, job grade, night and rotating shift work, overtime work, smoking, alcohol drinking, sleep duration, physical activity during work and housework or in commuting to work, and leisure-time physical activity. Physical activity during work and housework or in commuting and leisure time was expressed as the sum of metabolic equivalents (METs) multiplied by the duration of time (in hours) across physical activities of different levels. The psychological work environment was also assessed via the Japanese version of the Job Content Questionnaire [26], and the job strain score was calculated according to a standard procedure. Body height and weight were assessed to the nearest 0.1 cm and 0.1 kg, respectively, in a standardized procedure with participants wearing light clothes and without shoes. The body mass index was calculated as weight in kilograms divided by the square of height in meters (kg/m^2^). Dietary habits during the preceding one-month period were assessed using a validated brief self-administered diet history questionnaire (BDHQ) [27]. The dietary intake for energy and selected nutrients, including folate, vitamin B6, vitamin B12, n-3 polyunsaturated fatty acids, magnesium, and zinc, were estimated using an *ad hoc* computer algorithm for the BDHQ [28], with reference to the Standard Tables of Food Composition in Japan [29, 30].

### Statistical analyses

Differences in characteristics at the baseline survey between participants with and without depressive symptoms at the follow-up survey were assessed using the independent *t*-test (continuous variable) and the χ^2^-test (categorical variable). In addition, Pearson correlation coefficients across amino acid concentrations at baseline survey were calculated (n = 841). The association of baseline serum amino acids with subsequent development of depressive symptoms at the follow-up survey was evaluated by multiple logistic regression analysis (n = 841). We calculated the odds ratios (ORs) and 95% confidence intervals (CIs) of depressive symptoms for each tertile of serum amino acids at the baseline survey using the lowest tertile category as a reference. There is no consensus on optimal concentrations of circulating amino acids; we thus divided participants into the tertile of amino acid concentrations. We adjusted for age (year, continuous), sex, and site (surveyed in April 2015 or in May 2016) in the first model. The second model was further adjusted for marital status (married or other), job grade (low, middle, or high), night or rotating shift work (yes or no), overtime work (<10, 10–<30, or ≥30 h/month), job strain (quartile), physical activity at work and housework or while commuting to work (<3, 3–<7, 7–<20, or ≥20 METs-h/day), leisure-time physical activity (not engaged, >0–<3, 3–<10, or ≥10 METs-h/week), smoking habit (never-smoker, former smoker, current smoker consuming <20 cigarettes/day, or current smoker consuming ≥20 cigarettes/day), alcohol drinking habit (nondrinker, drinker consuming 1–3 days/month, weekly drinker consuming <1 go/day, 1 to <2 go/day, or ≥2 go/day; one go contains approximately 23 g of ethanol), sleep duration (<6, 6–6.9, or ≥7 h/day), body mass index (kg/m^2^, continuous), total energy intake (kcal/day, continuous), and intake of folate (μg/1000 kcal, continuous), vitamin B6 (mg/1000 kcal, continuous), vitamin B12 (μg/1000 kcal, continuous), n-3 polyunsaturated fatty acids (% energy, continuous), magnesium (mg/1000 kcal, continuous), and zinc (mg/1000 kcal, continuous). The nutritional factors we adjusted for have been linked to depressive symptoms [31–36]. We further adjusted for CES-D score. All models were adjusted for baseline values of potential confounding variables (described above). Trend associations were tested by assigning ordinal numbers (1–3) to tertile categories for each serum amino acid. To confirm the robustness of our findings, we repeated the above analysis after excluding participants whose blood sampling had not been performed after overnight fasting (n = 25). For other analyses, we considered a two-sided P <0.05 as statistically significant. All statistical analyses were performed using Stata, version 14.2 (StataCorp, College Station, TX, USA).

## Results

**Table 1** presents the baseline characteristics of the study populations according to their depressive symptoms at follow-up survey. Of the 841 participants free from depressive symptoms at baseline, 151 (18.0%) developed depressive symptoms (CES-D score ≥16) at follow-up survey. Participants with depressive symptom at the follow-up survey did not receive psychiatric treatment. Compared with participants free from depressive symptoms at the follow-up survey, those who developed depressive symptoms were younger and more likely to be in a low-ranking job position with a higher job strain and CES-D score but lower folate intake at baseline.

**Table 1:** Baseline characteristics of study population according to their depressive status at follow-up survey.

	Participants with depressive symptoms ^a^	Participants without depressive symptoms ^b^	P value ^c^
	(n = 151)	(n = 690)	
Age (mean ± s.d., year)	42.2 ± 8.1	44.8 ± 8.3	<0.001
Sex (women, %)	9.3	11.9	0.36
Site (survey in April 2015, %)	57.0	58.7	0.69
Marital status (married, %)	68.9	72.5	0.37
Job grade (low, %)	75.5	67.0	0.041
Night or rotating shift work (yes, %)	24.5	17.8	0.06
Overtime work (≥30 h/month, %)	27.2	27.3	0.98
Job strain (mean ± s.d.)	0.49 ± 0.10	0.47 ± 0.10	0.03
Physical activity at work and housework or while commuting to work (≥20 METs-h/day, %)	27.8	21.0	0.07
Leisure-time physical activity (≥10 METs-h/week, %)	23.8	28.6	0.24
Smoking habit (current, %)	33.1	25.7	0.06
Alcohol drinking habit (current ^d^, %)	47.7	54.8	0.11
Short sleep duration (<6 h/day, %)	51.7	52.0	0.93
BMI (mean ± s.d., kg/m^2^)	23.9 ± 4.0	23.6 ± 3.4	0.43
CES-D score (median, interquartile range)	12.0 (10.0–14.0)	9.0 (6.0–12.0)	<0.001
Daily dietary intake (mean ± s.d.)			
Total energy (kcal)	1890 ± 650	1797 ± 523	0.06
Magnesium (mg/1000 kcal)	126 ± 28	128 ± 27	0.24
Zinc (mg/1000 kcal)	4.2 ± 0.6	4.2 ± 0.7	0.73
Folate (μg/1000 kcal)	153 ± 57	164 ± 58	0.047
Vitamin B6 (mg/1000 kcal)	0.60 ± 0.16	0.62 ± 0.15	0.10
Vitamin B12 (μg /1000 kcal)	4.6 ± 2.5	4.6 ± 2.2	0.68
N-3 polyunsaturated fatty acids (% energy)	1.2 ± 0.4	1.2 ± 0.4	0.63
Protein (% energy)	13.9 ± 3.0	14.1 ± 2.6	0.62
Animal protein (% energy)	7.6 ± 3.0	7.8 ± 2.7	0.36
Plant protein (% energy)	6.4 ± 1.1	6.3 ± 1.1	0.26

Abbreviations: MET, metabolic equivalent; s.d., standard deviation.

^a^ Participants with a Center for Epidemiologic Studies Depression scale score ≥16.

^b^ Participants with a Center for Epidemiologic Studies Depression scale score <16.

^c^ For continuous variables, independent t test was used; for categorical variables, chi-square test was used.

^d^ Alcohol consumption of at least one day per week.

**Fig 2:**
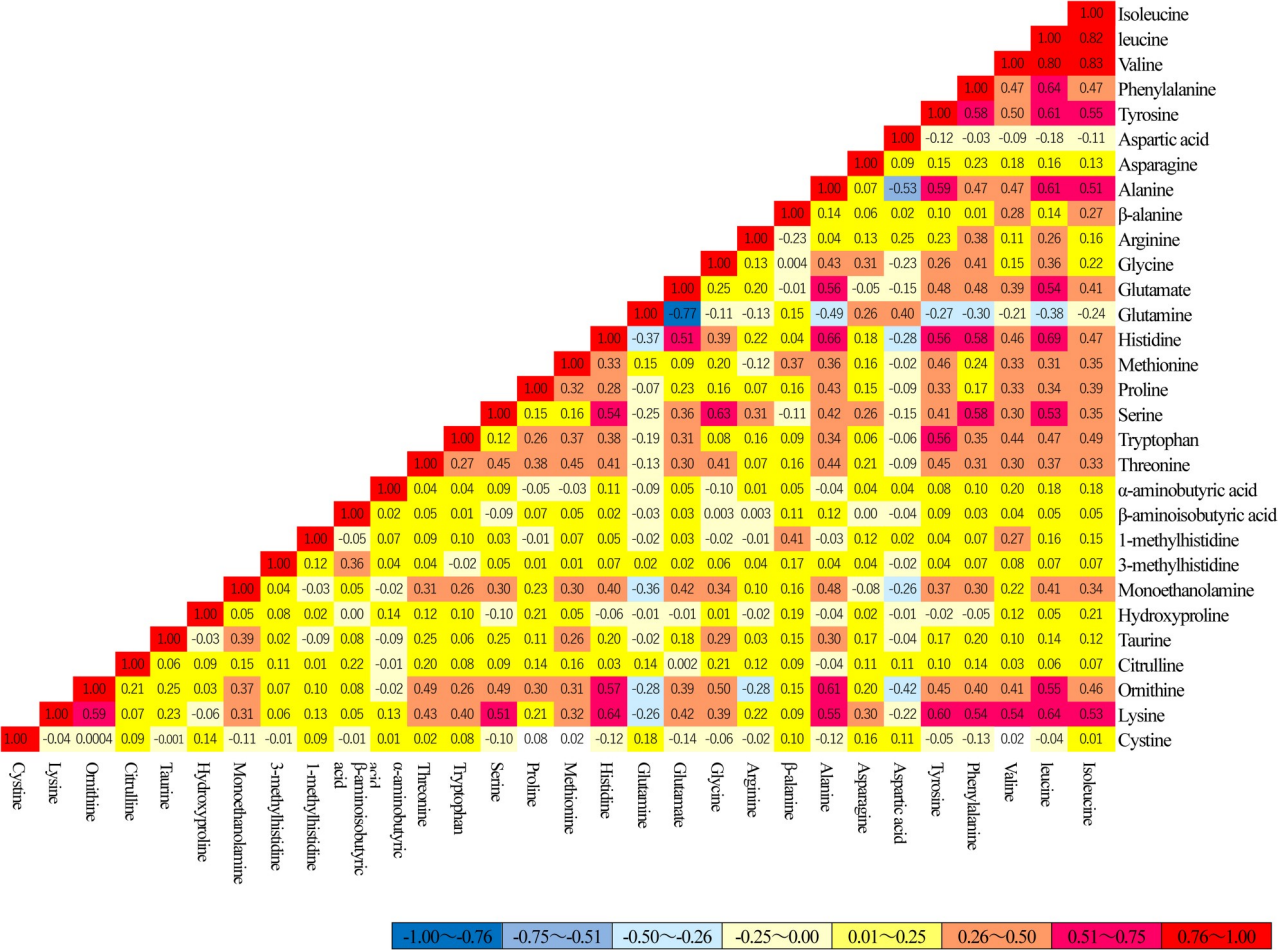
The correlations across serum amino acid concentrations at baseline. Abbreviation: CES-D, Center for Epidemiologic Studies Depression Scale.

The correlations among amino acid concentrations at baseline of the cohort are presented in **Fig 2**. Three BCAAs (isoleucine, leucine, and valine) were highly correlated with each other (r = 0.80–0.83), as well as glutamine and glutamate (r = −0.77). Other amino acids were not highly correlated with each other (r <0.64).

As shown in **Table 2**, there were no significant associations between tryptophan and glutamate concentrations and subsequent risk of depressive symptoms after adjusting for multiple confounders (model 2). The null association was robust when the analyses were performed after additional adjustment for baseline CES-D score (model 3) as well as after the exclusion of participants in whom blood sampling had not been performed after overnight fasting (model 4). Likewise, no significant associations were found for other amino acid concentrations (model 4) (**Tables 3–6**). Regarding serum arginine concentrations, a marginally significant trend was found for the risk of depressive symptoms after adjusting for multiple confounders (model 2) (P for trend = 0.09). The association were virtually unchanged after additional adjustment of baseline CES-D score (model 3); the multivariable-adjusted odds ratio for the highest versus lowest tertile of serum arginine was 1.65 (95% CI: 0.96–2.83; P for trend = 0.07). We confirmed similar findings after excluding participants whose blood sampling had not been performed after overnight fasting (model 4) (P for trend = 0.054).

**Table 2:** Associations of serum concentrations of gluconeogenic amino acids with depressive symptoms.

	Tertiles of serum concentrations of gluconeogenic amino acids			
	T1(low)	T2	T3 (high)	P for trend ^a^
**Aspartic acid (median, μmol/L)**	15.3	36.1	60.8	
Participants with/without depressive symptoms	50/231	50/232	51/227	
Model 1 ^b^	1.00 (ref)	1.09 (0.70–1.70)	1.28 (0.80–2.05)	0.30
Model 2 ^c^	1.00 (ref)	1.09 (0.69–1.74)	1.33 (0.81–2.18)	0.27
Model 3 ^d^	1.00 (ref)	1.05 (0.64–1.71)	1.25 (0.74–2.10)	0.41
Model 4 ^e^	1.00 (ref)	1.13 (0.68–1.88)	1.36 (0.79–2.33)	0.27
**Asparagine (median, μmol/L)**	45.8	57.6	69.1	
Participants with/without depressive symptoms	50/231	50/232	51/227	
Model 1 ^b^	1.00 (ref)	1.01 (0.65–1.55)	0.92 (0.59–1.43)	0.72
Model 2 ^c^	1.00 (ref)	0.96 (0.61–1.51)	0.82 (0.52–1.31)	0.41
Model 3 ^d^	1.00 (ref)	0.87 (0.54–1.40)	0.79 (0.48–1.29)	0.34
Model 4 ^e^	1.00 (ref)	0.90 (0.55–1.46)	0.77 (0.46–1.29)	0.33
**Alanine (median, μmol/L)**	556.2	676.6	820.6	
Participants with/without depressive symptoms	49/232	45/235	57/223	
Model 1 ^b^	1.00 (ref)	0.91 (0.57–1.44)	1.19 (0.76–1.88)	0.41
Model 2 ^c^	1.00 (ref)	0.84 (0.52–1.36)	1.07 (0.65–1.75)	0.71
Model 3 ^d^	1.00 (ref)	0.78 (0.47–1.29)	0.98 (0.58–1.64)	0.97
Model 4 ^e^	1.00 (ref)	0.80 (0.48–1.35)	0.90 (0.52–1.55)	0.72
**β-alanine (median, μmol/L)**	4.1	5.3	6.9	
Participants with/without depressive symptoms	48/242	48/219	52/216	
Model 1 ^b^	1.00 (ref)	1.10 (0.68–1.78)	1.13 (0.67–1.90)	0.64
Model 2 ^c^	1.00 (ref)	1.18 (0.71–1.95)	1.17 (0.67–2.02)	0.60
Model 3 ^d^	1.00 (ref)	1.17 (0.69–1.98)	1.19 (0.67–2.12)	0.57
Model 4 ^e^	1.00 (ref)	1.39 (0.80–2.40)	1.40 (0.76–2.58)	0.29
**Arginine (median, μmol/L)**	79.1	109.3	145.1	
Participants with/without depressive symptoms	43/238	55/224	53/227	
Model 1 ^b^	1.00 (ref)	1.09 (0.70–1.70)	1.28 (0.80–2.05)	0.30
Model 2 ^c^	1.00 (ref)	1.51 (0.94–2.43)	1.55 (0.93–2.58)	0.09
Model 3 ^d^	1.00 (ref)	1.58 (0.96–2.61)	1.65 (0.96–2.83)	0.07
Model 4 ^e^	1.00 (ref)	1.61 (0.95–2.71)	1.73 (0.99–3.04)	0.054
**Glycine (median, μmol/L)**	402.7	454.9	511.4	
Participants with/without depressive symptoms	45/236	47/233	59/221	
Model 1 ^b^	1.00 (ref)	1.07 (0.68–1.69)	1.44 (0.92–2.27)	0.10
Model 2 ^c^	1.00 (ref)	1.02 (0.64–1.65)	1.37 (0.86–2.20)	0.18
Model 3 ^d^	1.00 (ref)	0.94 (0.57–1.55)	1.38 (0.84–2.27)	0.19
Model 4 ^e^	1.00 (ref)	0.88 (0.53–1.48)	1.31 (0.78–2.18)	0.29
**Glutamate (median, μmol/L)**	303.6	379.4	496.5	
Participants with/without depressive symptoms	49/232	44/236	58/222	
Model 1 ^b^	1.00 (ref)	0.94 (0.59–1.50)	1.38 (0.86–2.21)	0.16
Model 2 ^c^	1.00 (ref)	1.03 (0.63–1.69)	1.51 (0.88–2.58)	0.13
Model 3 ^d^	1.00 (ref)	1.05 (0.62–1.78)	1.56 (0.88–2.76)	0.12
Model 4 ^e^	1.00 (ref)	1.06 (0.62–1.83)	1.52 (0.84–2.72)	0.16
**Glutamine (median, μmol/L)**	225.2	318.5	433.2	
Participants with/without depressive symptoms	50/230	57/221	43/236	
Model 1 ^b^	1.00 (ref)	1.20 (0.78–1.85)	0.82 (0.49–1.37)	0.56
Model 2 ^c^	1.00 (ref)	1.23 (0.78–1.95)	0.77 (0.43–1.35)	0.46
Model 3 ^d^	1.00 (ref)	1.18 (0.73–1.93)	0.72 (0.39–1.31)	0.37
Model 4 ^e^	1.00 (ref)	1.22 (0.73–2.03)	0.77 (0.42–1.44)	0.50
**Histidine (median, μmol/L)**	118.7	134.8	152.3	
Participants with/without depressive symptoms	45/240	51/225	55/225	
Model 1 ^b^	1.00 (ref)	1.22 (0.77–1.92)	1.29 (0.80–2.07)	0.30
Model 2 ^c^	1.00 (ref)	1.20 (0.74–1.92)	1.28 (0.77–2.10)	0.34
Model 3 ^d^	1.00 (ref)	1.18 (0.71–1.95)	1.32 (0.78–2.24)	0.30
Model 4 ^e^	1.00 (ref)	1.19 (0.71–1.99)	1.29 (0.74–2.23)	0.37
**Methionine (median, μmol/L)**	28.2	33.7	38.4	
Participants with/without depressive symptoms	52/230	47/234	52/226	
Model 1 ^b^	1.00 (ref)	0.87 (0.55–1.37)	0.97 (0.61–1.55)	0.91
Model 2 ^c^	1.00 (ref)	0.89 (0.55–1.43)	0.91 (0.54–1.50)	0.71
Model 3 ^d^	1.00 (ref)	0.88 (0.53–1.45)	0.95 (0.55–1.63)	0.87
Model 4 ^e^	1.00 (ref)	0.90 (0.53–1.52)	0.98 (0.56–1.71)	0.96
**Proline (median, μmol/L)**	165.9	202.5	247.0	
Participants with/without depressive symptoms	47/235	45/234	59/221	
Model 1 ^b^	1.00 (ref)	0.95 (0.60–1.50)	1.29 (0.83–2.01)	0.24
Model 2 ^c^	1.00 (ref)	0.99 (0.61–1.60)	1.30 (0.81–2.10)	0.27
Model 3 ^d^	1.00 (ref)	0.92 (0.55–1.52)	1.18 (0.72–1.96)	0.48
Model 4 ^e^	1.00 (ref)	0.89 (0.53–1.51)	1.12 (0.66–1.88)	0.65
**Serine (median, μmol/L)**	222.8	252.7	284.7	
Participants with/without depressive symptoms	44/238	47/232	60/220	
Model 1 ^b^	1.00 (ref)	1.06 (0.66–1.69)	1.55 (0.96–2.50)	0.07
Model 2 ^c^	1.00 (ref)	1.12 (0.69–1.81)	1.59 (0.95–2.66)	0.08
Model 3 ^d^	1.00 (ref)	1.19 (0.71–1.98)	1.54 (0.90–2.65)	0.12
Model 4 ^e^	1.00 (ref)	1.21 (0.72–2.04)	1.47 (0.84–2.59)	0.17
**Tryptophan (median, μmol/L)**	61.1	69.8	78.1	
Participants with/without depressive symptoms	44/238	42/237	65/215	
Model 1 ^b^	1.00 (ref)	0.91 (0.56–1.47)	1.52 (0.97–2.39)	0.051
Model 2 ^c^	1.00 (ref)	0.81 (0.49–1.35)	1.37 (0.84–2.22)	0.15
Model 3 ^d^	1.00 (ref)	0.90 (0.53–1.53)	1.34 (0.81–2.24)	0.21
Model 4 ^e^	1.00 (ref)	0.90 (0.52–1.55)	1.31 (0.77–2.23)	0.27
**Threonine (median, μmol/L)**	159.7	184.3	210.2	
Participants with/without depressive symptoms	49/232	54/226	48/232	
Model 1 ^b^	1.00 (ref)	1.06 (0.69–1.64)	0.97 (0.62–1.51)	0.88
Model 2 ^c^	1.00 (ref)	1.06 (0.67–1.67)	0.99 (0.62–1.59)	0.97
Model 3 ^d^	1.00 (ref)	0.96 (0.60–1.56)	0.92 (0.56–1.52)	0.74
Model 4 ^e^	1.00 (ref)	0.86 (0.52–1.41)	0.93 (0.56–1.56)	0.79

Abbreviations: ref, reference; T, tertile.

^a^ Based on multiple logistic regression analyses, assigning ordinal numbers of 1 to 3 to the tertile categories of the independent variable.

^b^ Adjusted for baseline values of age (year, continuous), sex, and site (survey in April 2015 or in May 2016).

^c^ Adjusted for baseline values of age (year, continuous), sex, site (survey in April 2015 or in May 2016), marital status (married or other), job grade (low, middle, or high), night or rotating shift work (yes or no), overtime work (<10, 10–<30, or ≥30 h/month), job strain (quartile), physical activity at work and housework or while commuting to work (<3, 3–<7, 7–<20, or ≥20 METs-h/day), leisure-time physical activity (not engaged, >0–<3, 3–<10, or ≥10 METs-h/week), smoking habit (never-smoker, former smoker, current smoker consuming <20 cigarettes/day, or current smoker consuming ≥20 cigarettes/day), alcohol drinking habit (nondrinker, drinker consuming 1–3 days/month, weekly drinker consuming <1 go/day, 1 to <2 go/day, or ≥2 go/day; one go contains approximately 23 g of ethanol), sleep duration (<6, 6–6.9, or ≥7 h/day), body mass index (kg/m^2^, continuous), total energy intake (kcal/day, continuous), and intake of folate (μg/1000 kcal, continuous), vitamin B6 (mg/1000 kcal, continuous), vitamin B12 (μg /1000 kcal, continuous), n-3 polyunsaturated fatty acids (% energy, continuous), magnesium (mg/1000 kcal, continuous), and zinc (mg/1000 kcal, continuous).

^d^ Model 2 + CES-D score at baseline (continuous) were further adjusted for.

^e^ Further excluded 25 participants whose blood sampling was not performed after over-night fasting.

**Table 3:** Associations of serum concentrations of branched-chain amino acids with depressive symptoms.

	Tertiles of serum concentrations of branched-chain amino acids			
	T1(low)	T2	T3 (high)	P for trend ^1^
**Isoleucine (median, μmol/L)**	80.7	95.0	109.7	
Participants with/without depressive symptoms	50/232	40/239	61/219	
Model 1 ^b^	1.00 (ref)	0.74 (0.46–1.19)	1.20 (0.76–1.89)	0.35
Model 2 ^c^	1.00 (ref)	0.68 (0.41–1.14)	1.09 (0.65–1.83)	0.61
Model 3 ^d^	1.00 (ref)	0.59 (0.34–1.01)	0.88 (0.50–1.53)	0.81
Model 4 ^e^	1.00 (ref)	0.52 (0.30–0.92)	0.83 (0.46–1.47)	0.67
**leucine (median, μmol/L)**	183.7	211.9	245.9	
Participants with/without depressive symptoms	53/228	38/242	60/220	
Model 1 ^b^	1.00 (ref)	0.64 (0.39–1.03)	1.09 (0.68–1.75)	0.62
Model 2 ^c^	1.00 (ref)	0.62 (0.36–1.07)	0.87 (0.49–1.52)	0.73
Model 3 ^d^	1.00 (ref)	0.90 (0.54–1.48)	1.56 (0.94–2.58)	0.06
Model 4 ^e^	1.00 (ref)	0.69 (0.39–1.21)	0.89 (0.50–1.61)	0.80
**Valine (median, μmol/L)**	252.1	291.7	332.5	
Participants with/without depressive symptoms	46/235	47/234	58/221	
Model 1 ^b^	1.00 (ref)	1.01 (0.64–1.61)	1.30 (0.82–2.06)	0.25
Model 2 ^c^	1.00 (ref)	0.96 (0.58–1.59)	1.20 (0.71–2.01)	0.46
Model 3 ^d^	1.00 (ref)	0.83 (0.49–1.42)	1.09 (0.63–1.88)	0.67
Model 4 ^e^	1.00 (ref)	0.87 (0.50–1.52)	1.16 (0.66–2.04)	0.55

Abbreviations: ref, reference; T, tertile.

^a^ Based on multiple logistic regression analyses, assigning ordinal numbers of 1 to 3 to the tertile categories of the independent variable.

^b^ Adjusted for baseline values of age (year, continuous), sex, and site (survey in April 2015 or in May 2016).

^c^ Adjusted for baseline values of age (year, continuous), sex, site (survey in April 2015 or in May 2016), marital status (married or other), job grade (low, middle, or high), night or rotating shift work (yes or no), overtime work (<10, 10–<30, or ≥30 h/month), job strain (quartile), physical activity at work and housework or while commuting to work (<3, 3–<7, 7–<20, or ≥20 METs-h/day), leisure-time physical activity (not engaged, >0–<3, 3–<10, or ≥10 METs-h/week), smoking habit (never-smoker, former smoker, current smoker consuming <20 cigarettes/day, or current smoker consuming ≥20 cigarettes/day), alcohol drinking habit (nondrinker, drinker consuming 1–3 days/month, weekly drinker consuming <1 go/day, 1 to <2 go/day, or ≥2 go/day; one go contains approximately 23 g of ethanol), sleep duration (<6, 6–6.9, or ≥7 h/day), body mass index (kg/m^2^, continuous), total energy intake (kcal/day, continuous), and intake of folate (μg/1000 kcal, continuous), vitamin B6 (mg/1000 kcal, continuous), vitamin B12 (μg /1000 kcal, continuous), n-3 polyunsaturated fatty acids (% energy, continuous), magnesium (mg/1000 kcal, continuous), and zinc (mg/1000 kcal, continuous).

^d^ Model 2 + CES-D score at baseline (continuous) were further adjusted for.

^e^ Further excluded 25 participants whose blood sampling was not performed after over-night fasting.

**Table 4:** Associations of serum concentrations of aromatic amino acids with depressive symptoms.

	Tertiles of serum concentrations of aromatic amino acids			
	T1(low)	T2	T3 (high)	P for trend ^a^
**Phenylalanine (median, μmol/L)**	116.4	131.2	148.2	
Participants with/without depressive symptoms	54/228	40/240	57/222	
Model 1 ^b^	1.00 (ref)	0.76 (0.48–1.21)	1.21 (0.77–1.89)	0.40
Model 2 ^c^	1.00 (ref)	0.77 (0.47–1.25)	1.09 (0.66–1.79)	0.73
Model 3 ^d^	1.00 (ref)	0.70 (0.42–1.16)	1.11 (0.65–1.88)	0.71
Model 4 ^e^	1.00 (ref)	0.72 (0.43–1.22)	1.08 (0.62–1.86)	0.80
**Tyrosine (median, μmol/L)**	67.5	78.9	91.5	
Participants with/without depressive symptoms	48/235	41/237	62/218	
Model 1 ^b^	1.00 (ref)	0.86 (0.54–1.38)	1.46 (0.93–2.28)	0.08
Model 2 ^c^	1.00 (ref)	0.78 (0.47–1.28)	1.34 (0.81–2.24)	0.22
Model 3 ^d^	1.00 (ref)	0.66 (0.39–1.12)	1.12 (0.65–1.92)	0.59
Model 4 ^e^	1.00 (ref)	0.69 (0.40–1.18)	1.10 (0.63–1.94)	0.66

Abbreviations: ref, reference; T, tertile.

^a^ Based on multiple logistic regression analyses, assigning ordinal numbers of 1 to 3 to the tertile categories of the independent variable.

^b^ Adjusted for baseline values of age (year, continuous), sex, and site (survey in April 2015 or in May 2016).

^c^ Adjusted for baseline values of age (year, continuous), sex, site (survey in April 2015 or in May 2016), marital status (married or other), job grade (low, middle, or high), night or rotating shift work (yes or no), overtime work (<10, 10–<30, or ≥30 h/month), job strain (quartile), physical activity at work and housework or while commuting to work (<3, 3–<7, 7–<20, or ≥20 METs-h/day), leisure-time physical activity (not engaged, >0–<3, 3–<10, or ≥10 METs-h/week), smoking habit (never-smoker, former smoker, current smoker consuming <20 cigarettes/day, or current smoker consuming ≥20 cigarettes/day), alcohol drinking habit (nondrinker, drinker consuming 1–3 days/month, weekly drinker consuming <1 go/day, 1 to <2 go/day, or ≥2 go/day; one go contains approximately 23 g of ethanol), sleep duration (<6, 6–6.9, or ≥7 h/day), body mass index (kg/m^2^, continuous), total energy intake (kcal/day, continuous), and intake of folate (μg/1000 kcal, continuous), vitamin B6 (mg/1000 kcal, continuous), vitamin B12 (μg /1000 kcal, continuous), n-3 polyunsaturated fatty acids (% energy, continuous), magnesium (mg/1000 kcal, continuous), and zinc (mg/1000 kcal, continuous).

^d^ Model 2 + CES-D score at baseline (continuous) were further adjusted for.

^e^ Further excluded 25 participants whose blood sampling was not performed after over-night fasting.

**Table 5:** Associations of intermediary organic acids with depressive symptoms.

	Tertiles of serum concentrations of intermediary organic acids			
	T1(low)	T2	T3 (high)	P for trend ^a^
α-aminobutyric acid (median, μmol/L)	14.1	18.8	25.0	
Participants with/without depressive symptoms	54/228	49/230	48/232	
Model 1 ^b^	1.00 (ref)	0.87 (0.56–1.33)	0.91 (0.59–1.40)	0.65
Model 2 ^c^	1.00 (ref)	0.96 (0.60–1.53)	1.16 (0.72–1.89)	0.55
Model 3 ^d^	1.00 (ref)	0.96 (0.59–1.56)	1.16 (0.70–1.93)	0.56
Model 4 ^e^	1.00 (ref)	0.90 (0.54–1.49)	1.24 (0.73–2.10)	0.43
β-aminoisobutyric acid (median, μmol/L)	3.3	4.1	5.10	
Participants with/without depressive symptoms	19/74	9/71	13/71	
Model 1 ^b^	1.00 (ref)	0.48 (0.20–1.15)	0.80 (0.36–1.78)	0.51
Model 2 ^c^	1.00 (ref)	0.18 (0.05–0.64)	0.39 (0.12–1.28)	0.12
Model 3 ^d^	1.00 (ref)	1.18 (0.71–1.95)	1.17 (0.67–2.02)	0.60
Model 4 ^e^	1.00 (ref)	0.17 (0.04–0.65)	0.43 (0.13–1.47)	0.18
1-methylhistidine (median, μmol/L)	5.0	8.0	15.7	
Participants with/without depressive symptoms	31/104	18/117	27/108	
Model 1 ^b^	1.00 (ref)	0.50 (0.26–0.95)	0.79 (0.44–1.42)	0.41
Model 2 ^c^	1.00 (ref)	0.48 (0.23–0.99)	0.75 (0.39–1.47)	0.40
Model 3 ^d^	1.00 (ref)	0.48 (0.23–1.02)	0.79 (0.39–1.59)	0.48
Model 4 ^e^	1.00 (ref)	0.52 (0.24–1.13)	0.89 (0.42–1.86)	0.72
3-methylhistidine (median, μmol/L)	4.2	4.7	5.7	
Participants with/without depressive symptoms	29 /150	26 /124	31/119	
Model 1 ^b^	1.00 (ref)	1.06 (0.59–1.90)	1.28 (0.73–2.26)	0.39
Model 2 ^c^	1.00 (ref)	1.00 (0.54–1.88)	1.20 (0.64–2.23)	0.58
Model 3 ^d^	1.00 (ref)	0.92 (0.47–1.80)	1.28 (0.66–2.48)	0.49
Model 4 ^e^	1.00 (ref)	0.92 (0.46–1.83)	1.16 (0.58–2.31)	0.70
**Monoethanolamine (median, μmol/L)**	22.0	25.3	28.8	
Participants with/without depressive symptoms	57/228	46/232	48/230	
Model 1 ^b^	1.00 (ref)	0.76 (0.49–1.18)	0.79 (0.51–1.23)	0.30
Model 2 ^c^	1.00 (ref)	0.79 (0.51–1.25)	0.83 (0.52–1.33)	0.43
Model 3 ^d^	1.00 (ref)	0.81 (0.50–1.30)	0.79 (0.48–1.29)	0.33
vModel 4 ^e^	1.00 (ref)	0.80 (0.49–1.32)	0.77 (0.46–1.28)	0.31
**Hydroxyproline (median, μmol/L)**	11.0	13.5	18.4	
Participants with/without depressive symptoms	33/163	48/144	33/163	
Model 1 ^b^	1.00 (ref)	1.57 (0.95–2.60)	0.92 (0.53–1.59)	0.80
Model 2 ^c^	1.00 (ref)	1.58 (0.92–2.72)	0.85 (0.48–1.53)	0.58
Model 3 ^d^	1.00 (ref)	1.77 (0.996–3.15)	0.93 (0.51–1.70)	0.80
Model 4 ^e^	1.00 (ref)	1.64 (0.90–3.00)	0.89 (0.47–1.67)	0.70
**Taurine (median, μmol/L)**	191.0	220.4	255.4	
Participants with/without depressive symptoms	52/230	48/231	51/229	
Model 1 ^b^	1.00 (ref)	0.92 (0.59–1.42)	0.99 (0.59–0.64)	0.97
Model 2 ^c^	1.00 (ref)	0.90 (0.58–1.42)	0.90 (0.57–1.42)	0.64
Model 3 ^d^	1.00 (ref)	0.97 (0.60–1.56)	0.91 (0.56–1.47)	0.70
Model 4 ^e^	1.00 (ref)	1.03 (0.63–1.69)	0.91 (0.55–1.50)	0.72

Abbreviations: ref, reference; T, tertile.

^a^ Based on multiple logistic regression analyses, assigning ordinal numbers of 1 to 3 to the tertile categories of the independent variable.

^b^ Adjusted for baseline values of age (year, continuous), sex, and site (survey in April 2015 or in May 2016).

^c^ Adjusted for baseline values of age (year, continuous), sex, site (survey in April 2015 or in May 2016), marital status (married or other), job grade (low, middle, or high), night or rotating shift work (yes or no), overtime work (<10, 10–<30, or ≥30 h/month), job strain (quartile), physical activity at work and housework or while commuting to work (<3, 3–<7, 7–<20, or ≥20 METs-h/day), leisure-time physical activity (not engaged, >0–<3, 3–<10, or ≥10 METs-h/week), smoking habit (never-smoker, former smoker, current smoker consuming <20 cigarettes/day, or current smoker consuming ≥20 cigarettes/day), alcohol drinking habit (nondrinker, drinker consuming 1–3 days/month, weekly drinker consuming <1 go/day, 1 to <2 go/day, or ≥2 go/day; one go contains approximately 23 g of ethanol), sleep duration (<6, 6–6.9, or ≥7 h/day), body mass index (kg/m^2^, continuous), total energy intake (kcal/day, continuous), and intake of folate (μg/1000 kcal, continuous), vitamin B6 (mg/1000 kcal, continuous), vitamin B12 (μg /1000 kcal, continuous), n-3 polyunsaturated fatty acids (% energy, continuous), magnesium (mg/1000 kcal, continuous), and zinc (mg/1000 kcal, continuous).

^d^ Model 2 + CES-D score at baseline (continuous) were further adjusted for.

^e^ Further excluded 25 participants whose blood sampling was not performed after over-night fasting.

**Table 6:** Associations of serum concentrations of other amino acids with depressive symptoms.

	Tertiles of serum concentrations of other amino acids			
	T1(low)	T2	T3 (high)	P for trend ^a^
**Citrulline (median, μmol/L)**	25.7	32.2	39.4	
Participants with/without depressive symptoms	48/235	52/227	51/228	
Model 1 ^b^	1.00 (ref)	1.18 (0.76–1.83)	1.24 (0.79–1.94)	0.35
Model 2 ^c^	1.00 (ref)	1.16 (0.73–1.85)	1.20 (0.74–1.93)	0.46
Model 3 ^d^	1.00 (ref)	1.16 (0.71–1.90)	1.09 (0.66–1.81)	0.73
Model 4 ^e^	1.00 (ref)	1.29 (0.77–2.14)	1.12 (0.66–1.89)	0.68
**Ornithine (median, μmol/L)**	144.6	188.4	230.0	
Participants with/without depressive symptoms	44/237	49/231	58/222	
Model 1 ^b^	1.00 (ref)	1.08 (0.67–1.74)	1.39 (0.86–2.23)	0.16
Model 2 ^c^	1.00 (ref)	0.96 (0.58–1.59)	1.15 (0.69–1.91)	0.56
Model 3 ^d^	1.00 (ref)	0.85 (0.50–1.45)	1.05 (0.61–1.81)	0.77
Model 4 ^e^	1.00 (ref)	0.86 (0.49–1.48)	1.002 (0.57–1.75)	0.94
**Lysine (median, μmol/L)**	235.4	279.0	317.0	
Participants with/without depressive symptoms	45/237	41/238	65/215	
Model 1 ^b^	1.00 (ref)	0.92 (0.57–1.48)	1.65 (1.03–2.63)	0.03
Model 2 ^c^	1.00 (ref)	0.90 (0.54–1.48)	1.56 (0.94–2.58)	0.06
Model 3 ^d^	1.00 (ref)	0.79 (0.46–1.34)	1.38 (0.81–2.35)	0.17
Model 4 ^e^	1.00 (ref)	0.77 (0.44–1.33)	1.44 (0.83–2.50)	0.13
**Cystine (median, μmol/L)**	2.6	3.9	6.5	
Participants with/without depressive symptoms	38/207	43/178	43/185	
Model 1 ^b^	1.00 (ref)	1.37 (0.84–2.22)	1.28 (0.79–2.08)	0.31
Model 2 ^c^	1.00 (ref)	1.50 (0.90–2.51)	1.32 (0.79–2.22)	0.28
Model 3 ^d^	1.00 (ref)	1.93 (1.11–3.34)	1.54 (0.89–2.66)	0.12
Model 4 ^e^	1.00 (ref)	1.93 (1.10–3.38)	1.53 (0.88–2.69)	0.13

Abbreviations: ref, reference; T, tertile.

^a^ Based on multiple logistic regression analyses, assigning ordinal numbers of 1 to 3 to the tertile categories of the independent variable.

^b^ Adjusted for baseline values of age (year, continuous), sex, and site (survey in April 2015 or in May 2016).

^c^ Adjusted for baseline values of age (year, continuous), sex, site (survey in April 2015 or in May 2016), marital status (married or other), job grade (low, middle, or high), night or rotating shift work (yes or no), overtime work (<10, 10–<30, or ≥30 h/month), job strain (quartile), physical activity at work and housework or while commuting to work (<3, 3–<7, 7–<20, or ≥20 METs-h/day), leisure-time physical activity (not engaged, >0–<3, 3–<10, or ≥10 METs-h/week), smoking habit (never-smoker, former smoker, current smoker consuming <20 cigarettes/day, or current smoker consuming ≥20 cigarettes/day), alcohol drinking habit (nondrinker, drinker consuming 1–3 days/month, weekly drinker consuming <1 go/day, 1 to <2 go/day, or ≥2 go/day; one go contains approximately 23 g of ethanol), sleep duration (<6, 6–6.9, or ≥7 h/day), body mass index (kg/m^2^, continuous), total energy intake (kcal/day, continuous), and intake of folate (μg/1000 kcal, continuous), vitamin B6 (mg/1000 kcal, continuous), vitamin B12 (μg /1000 kcal, continuous), n-3 polyunsaturated fatty acids (% energy, continuous), magnesium (mg/1000 kcal, continuous), and zinc (mg/1000 kcal, continuous).

^d^ Model 2 + CES-D score at baseline (continuous) were further adjusted for.

^e^ Further excluded 25 participants whose blood sampling was not performed after over-night fasting.

## Discussion

In this prospective study of Japanese employees, we found no significant association between tryptophan or glutamate concentrations and subsequent risk of depressive symptoms after adjustment for a wide range of dietary and lifestyle factors. For serum arginine, a marginally significant trend was found for risk of depressive symptoms. To our knowledge, this is the first prospective study to investigate the association between serum amino acid profiles and depressive status.

Tryptophan, an essential amino acid, is a precursor of serotonin synthesis, which is thought to be involved in the pathophysiology of depression [37]. Consistent with this pathophysiological evidence, a meta-analysis indicated that patients with depressive disorder had significantly lower plasma tryptophan concentrations than healthy controls [10]. Contrary to our expectation, serum tryptophan concentrations were not associated with subsequent risk of depressive symptoms in the present cohort. This null finding is consistent with those of two cross-sectional studies among the community-dwelling elderly in Japan [21] and in Mexico [17]. Results of these population-based studies including ours do not support the hypothesis that higher circulating concentrations of tryptophan are associated with a prevalence or risk of depressive symptoms.

Mechanistic evidence suggests that glutamate system plays a major role in the pathogenesis of depressive disorders [38], which has been supported by a meta-analysis that showed significantly higher blood glutamate concentrations in patients with major depressive disorder than in controls [11]. In the present study, however, we found no measurable relationship between serum glutamate concentrations and subsequent risk of depressive symptoms. Our findings agreed with those of a cross-sectional study of the free-living elderly in Japan [21], which found no association, but contradict with the results of another cross-sectional study of community-dwelling elderly women in Mexico, which showed that depressed individuals had an increased plasma glutamate concentrations compared with control individuals [17]. Given these data, we speculate that alternation of circulating glutamate concentrations in depressive symptoms might be less sensitive for Asian populations. Further investigations will be necessary to support or refute this assumption.

We found a marginally significant trend of an increased risk of depressive symptoms with increasing serum arginine concentrations. To date, a cross-sectional study of community-dwelling elderly women in Mexico found that depressive patients had a significantly higher concentrations of arginine than healthy controls [17], whereas another cross-sectional study of free-living elderly in Japan found no measurable association between them [21]. Similarly, clinical studies found inconsistent results. There were no significant differences in the plasma concentrations of arginine between depressive patients and healthy control participants [14, 18, 39] or even significantly lowered serum concentrations of arginine in patients with depression compared with controls [40]. Pathophysiological evidence suggests that higher concentrations of nitric oxide (NO), which is synthesized from arginine, are neurotoxic by mediating neuroinflammatory actions [41]. In addition, experimental evidence from an animal study revealed that NO synthase inhibitors enhanced the extracellular level of serotonin and dopamine in the rat ventral hippocampus, whereas the NO precursor arginine had the opposite effect [42]. Another study also added biological plausibility to the neurotoxic properties of arginine by showing that dietary arginine depletion reduced depressive-like response in male but not female mice [43]. Further researches are needed to determine whether higher concentrations of circulating arginine concentrations are associated with an increased risk of depressive symptoms.

Here, we discuss our null findings of circulating amino acid concentrations and the risk of depressive symptoms in the context of brain amino concentrations. In most brain regions, the uptake of circulating amino acids is suggested to be limited by the blood-brain barrier (BBB) and thus amino acids concentrations in brain are maintained fairly independently of those of circulating concentrations [44, 45]. Meanwhile, it is speculated that peripheral amino acids alteration might, to some extent, reflect those changes observed in the brain [46]. Studies that measured both peripheral and brain amino acid concentrations showed inconsistent data. For instance, the correlation between central and peripheral tryptophan concentrations was moderate in some (r = 0.47) [47] but poor in other studies (r = 0.14–0.15) [48, 49]. Likewise, the correlation between central and peripheral glutamate concentrations was moderate in some (r = 0.67) [50] but poor in other studies (r = 0.02) [51]. To date, depression studies that measured brain amino concentrations have also shown mixed results. A study found significantly lower brain tryptophan concentrations in depressive patients compared with control [52], while another study showed no significant difference between those groups [53]. Similarly, some studies observed significantly higher brain glutamate concentrations in depressed participants compared with controls [54, 55], whereas other studies showed no difference [56, 57] or even decreased of glutamate concentrations in patients with depression compared with controls [58]. Given these inconsistency regarding the correlation of amino acid concentrations between blood and brain and the relationship of central amino acid concentrations with depression, further studies are required to clarify the significance of circulating forms of amino acids in the development of depression.

The strengths of this study include its prospective design, use of validated measurements for nutrient intake (i.e., the BDHQ) as well as depressive status (i.e., CES-D score), and adjustment for various dietary and lifestyle factors. In addition, as the study survey was carried out in a company during a periodic health check-up and had a high participation rate, the possibility of bias linked to selective survey participation is low. We also acknowledge limitations of our study. First, 32.7% did not take part in the follow-up survey, which may lead to selection bias. Second, serum amino acids might be less stable than those in plasma, but serum samples provide more sensitive results for amino acid detection [11, 59]. Third, since we made numerous tests, chance may explain some of the significant associations. The interpretation of the present findings thus requires caution. Fourth, we used a validated questionnaire (i.e. the CES-D scale) to assess depressive symptoms rather than structured diagnostic interviews. Thus, the findings may have differed if the outcome was defined by clinically diagnosed depression. Fifth, although we considered various potential confounders, we cannot rule out the possibility of bias due to unmeasured or residual confounding factors. Finally, our study was carried out among employees of a Japanese manufacturing company, hence caution is required when generalizing the present findings.

## Conclusion

We observed no prospective association between serum amino acid concentrations and depressive symptoms among Japanese workers. Few prospective studies have investigated this association, and further prospective studies among populations with different ethnicity, gender, age, and physiological and psychological conditions are warranted to assess these associations.
